# Supplementary material for: Effects of COVID-19 Financial and Social Hardships on Infants’ and Toddlers’ Development in the ECHO Program
Source: Int J Environ Res Public Health. 2023 Jan 5;20(2):1013. doi: 10.3390/ijerph20021013 (PMC9858743; doi:10.3390/ijerph20021013)
Supplement: Supplementary file 1 [file ijerph-20-01013-s001.zip › ijerph-2027561-supplementary.pdf]

Supplementary Materials

Table S1. Results of Univariate Models Testing For the Effects Of COVID-Related Hardships on Change in ASQ Category Scores.

	**Communication**		**Gross Motor**		**Fine Motor**		**Problem-solving**		**Personal-Social**	
	**OR (95%CI)**	**P value**	**OR (95%CI)**	**P value**	**OR (95%CI)**	**P value**	**OR (95%CI)**	P value	OR (95%CI)	P value
**Pandemic-Related Hardship**										
Parents' job loss/change	1.14 (0.72,2)	0.58	0.88 (0.53,1)	0.62	0.72 (0.42,1)	0.24	1.13 (0.58,2)	0.72	1.11 (0.64,2)	0.72
Less contact with household members	0.92 (0.52,2)	0.78	0.8 (0.43,1)	0.49	1.35 (0.74,2)	0.33	0.58 (0.25,1)	**0.18**	0.77 (0.37,2)	0.48
Less contact with family/friends outside	**1.64 (0.79,3)**	0.18	0.85 (0.43,2)	0.64	0.66 (0.34,1)	0.21	0.92 (0.39,2)	0.85	1.22 (0.56,3)	**0.61**
**Pre-Pandemic ASQ Scores**										
Pre-pandemic ASQ z-score	**0.84 (0.67,1)**	0.11	0.87 (0.66,1)	0.32	0.77 (0.6,1)	**0.04**	0.79 (0.58,1)	**0.14**	0.82 (0.63,1)	**0.12**
**Maternal Characteristics**										
**Maternal Race (Reference: white)**										
American Indian or Alaska Native	1.16 (0.51,3)	0.73	1.26 (0.56,3)	0.58	0.87 (0.35,2)	0.76	1.74 (0.7,4)	0.23	1.02 (0.42,2)	0.97
Asian	0.86 (0.45,2)	0.64	1.01 (0.5,2)	0.98	1.23 (0.6,3)	0.56	1.58 (0.66,4)	0.30	1.59 (0.79,3)	0.20
Black	1.47 (0.58,4)	0.42	1.66 (0.69,4)	0.26	1.44 (0.37,6)	0.60	2.12 (0.56,8)	0.27	1.09 (0.31,4)	0.90
Multiple race	1.55 (0.66,4)	0.32	0.39 (0.09,2)	0.20	0.88 (0.25,3)	0.84	1.33 (0.37,5)	0.67	1.5 (0.56,4)	0.42
Native Hawaiian or other Pacific Islander	**4.57 (0.93,22)**	0.06	**3.24(0.58,18)**	0.18	14 (1.22,160)	**0.03**	5.25 (0.45,62)	**0.19**	3.62 (0.33,39)	0.29
Other race	**2.59 (1.6,4)**	0.00	0.77 (0.38,2)	0.47	1.09 (0.58,2)	0.79	1.53 (0.71,3)	0.28	1.08 (0.55,2)	0.82
**Maternal Ethnicity**										
Not Hispanic or Latino vs Hispanic	**0.43 (0.29,1)**	0.00	1.09 (0.67,2)	0.74	0.85 (0.52,1)	0.52	0.63 (0.36,1)	**0.12**	0.84 (0.51,1)	0.49

Maternal Education (References: College degree)

Less than High School	**2.81 (1.35,6)**	0.01	1.11 (0.42,3)	0.83	3.24 (1.45,7)	**0.00**	3.15 (1.18,8)	**0.02**	0.85 (0.3,2)	0.76
High school degree, GED or equivalent	2.87 (1.57,5)	0.00	1.55 (0.76,3)	0.23	2.77 (1.3,6)	0.01	3.99 (1.7,9)	0.00	1.21 (0.55,3)	0.64
Some College, no Degree, or AA	1.37 (0.75,2)	0.31	0.53 (0.25,1)	0.09	0.85 (0.36,2)	0.70	0.87 (0.33,2)	0.78	0.78 (0.37,2)	0.50
Master's Degree, Professional or Doctorate Degree	0.71 (0.41,1)	0.23	0.86 (0.5,1)	0.60	0.98 (0.49,2)	0.96	1.31 (0.59,3)	0.51	1.11 (0.61,2)	0.74
**Maternal Employment**: No vs Yes	1.18 (0.46,3)	0.72	1.38 (0.62,3)	0.43	1.24 (0.36,4)	0.72	1.77 (0.66,5)	0.25	0.86 (0.28,3)	0.78
**Household income (References: > $100,000)**										
<$30,000	3.04 (1.87,5)	0.00	1.2 (0.68,2)	0.53	1.83 (0.98,3)	0.06	2.45 (1.21,5)	0.01	1.21 (0.68,2)	0.52
$30,000-49,999	**1.68 (0.79,4)**	0.18	0.71 (0.27,2)	0.49	1.37 (0.54,3)	0.50	1.36 (0.45,4)	0.59	1.17 (0.5,3)	0.72
$50,000- 74,999	**2.06 (0.99,4)**	0.05	0.83 (0.35,2)	0.68	1.58 (0.63,4)	0.33	1.63 (0.49,5)	0.43	1.44 (0.55,4)	0.45
$75,000-99,999	1.1 (0.47,3)	0.82	1.13 (0.49,3)	0.77	1.28 (0.54,3)	0.58	1.15 (0.34,4)	0.82	0.82 (0.32,2)	0.68
Marital Status	1.29 (0.69,2)	0.42	1.23 (0.6,2)	0.57	1.77 (0.83,4)	0.14	1.83 (0.81,4)	**0.15**	0.84 (0.34,2)	0.71
**Child Characteristics**										
Preterm: 37+wks vs < 37wks	0.91 (0.47,2)	0.78	1.01 (0.46,2)	0.98	0.77 (0.39,2)	0.47	1.13 (0.42,3)	0.81	1.92 (0.79,5)	**0.15**
Children Sex: Male vs Female	**2.44 (1.61,4)**	0.00			1.32 (0.81,2)	0.26	1.58 (0.9,3)	**0.11**	2.4 (1.46,4)	**0.00**
Children's Race (Reference: White)										
Black	1.32 (0.52,3)	0.56	1.18 (0.43,3)	0.75	1.28 (0.27,6)	0.75	1.54 (0.36,7)	0.56	1.04 (0.27,4)	0.95
Other	0.8 (0.51,1)	0.33	0.88 (0.53,1)	0.61	0.89 (0.52,2)	0.67	1.26 (0.7,2)	0.44	1.04 (0.61,2)	0.87
Children Ethnicity: Not Hispanic vs Hispanic	**0.42 (0.28,1)**	0.00	1.17 (0.72,2)	0.52	0.78 (0.48,1)	0.30	0.66 (0.37,1)	**0.15**	0.93 (0.57,2)	0.76
Gestational age	0.98 (0.92,1)	0.59	0.97 (0.91,1)	0.45	1.01 (0.93,1)	0.83	1.05 (0.91,1)	0.53	1.1 (0.97,1)	**0.13**
Birth weight	1 (1,1)	0.85	**1 (1,1)**	0.17	1 (1,1)	0.28	1 (1,1)	0.37	1 (1,1)	0.21

Children Pre-Pandemic Life Stage (reference: infancy)	-0.79 (-0.54,-1)	0.23	-1.19 (-0.76,-2)	0.44	-1.05(-0.65,-2)	0.83	-1.42 (-0.81,-2)	0.22	-0.66 (-0.4,-1)	**0.10**

Note. OR= Odds Ratio and CI = Confidence Interval; ASQ (Ages and Stages Questionnaire); Values in bold indicates covariates with p-values smaller than .2.

Table S2. Results of Multivariate Models Testing For The Effects Of COVID-Related Hardships on ASQ Categories (adjusted for pre-pandemic ASQ Scores and Significant Covariates (p-values <.2).

	**Parents' job loss/change**			**Less contact with household members**			**Less contact with family/friends outside home**		
	Gross Motor	Fine motor	Problem solving	Gross Motor	Fine motor	Problem solving	Gross Motor	Fine motor	Problem solving
	OR (95%CI)	OR (95%CI)	OR (95%CI)	OR (95%CI)	OR (95%CI)	OR (95%CI)	OR (95%CI)	OR (95%CI)	OR (95%CI)
Parents' job loss/change	0.86 (0.48,2)	0.5 (0.26,1)	0.75 (0.34,2)						
Less contact with household members				0.88 (0.47,2)	1.48 (0.74,3)	0.55 (0.22,1)			
Less contact with family/friends outside home							0.86 (0.44,2)	0.73 (0.36,1)	1.06 (0.45,2)
Baseline ASQ	0.76 (0.55,1)	0.66 (0.48,1)	0.97 (0.68,1)	1.16 (0.94,1)	1.08 (0.89,1)	1.17 (0.93,1)	1.16 (0.94,1)	1.09 (0.9,1)	1.14 (0.92,1)
**Maternal Characteristics**									
American Indian or Alaska Native	1.12 (0.43,3)	0.39 (0.1,1)	1.18 (0.25,6)	1.2 (0.49,3)	0.27 (0.08,1)	0.96 (0.27,3)	1.13 (0.47,3)	0.29 (0.09,1)	0.83 (0.24,3)
Asian	0.97 (0.42,2)	0.99 (0.38,3)	2 (0.7,6)	0.94 (0.46,2)	1.23 (0.53,3)	2 (0.8,5)	0.93 (0.46,2)	1.32 (0.59,3)	1.86 (0.75,5)
Black	1.98 (0.69,6)	0.36 (0.06,2)	0.7 (0.09,5)	1.51 (0.55,4)	0.56 (0.14,2)	1.01 (0.23,4)	1.51 (0.56,4)	0.55 (0.14,2)	1.02 (0.22,5)

Multiple race	0.57 (0.13,2)	1.19 (0.32,4)	1.68 (0.41,7)	0.4 (0.09,2)	0.77 (0.25,2)	1.14 (0.31,4)	0.39 (0.09,2)	0.76 (0.24,2)	1.12 (0.3,4)
Native Hawaiian or other Pacific Islander	2.53 (0.47,13)	4.36 (0.24,79)	1.69 (0.07,41)	2.11 (0.35,13)	1.49 (0.17,13)	0.81 (0.06,12)	2.17 (0.36,13)	1.59 (0.18,14)	0.8 (0.05,12)
Other race	0.42 (0.16,1)	0.43 (0.13,1)	0.36 (0.07,2)	0.61 (0.29,1)	0.32 (0.12,1)	0.45 (0.14,2)	0.61 (0.29,1)	0.33 (0.13,1)	0.44 (0.13,1)
Maternal ethnicity			0.47 (0.17,1)			0.71 (0.29,2)			0.7 (0.29,2)
Less than high school	1.9 (0.45,8)	7.66 (1.73,34)	1.72 (0.24,12)	1.32 (0.47,4)	8.66 (2.61,29)	3.05 (0.66,14)	1.32 (0.48,4)	8.22 (2.49,27)	3.24 (0.68,15)
High school degree, GED or equivalent	1.54 (0.52,5)	3.49 (0.84,15)	1.71 (0.34,9)	1.57 (0.7,4)	**5.4 (1.71,17)**	3.74 (0.94,15)	1.56 (0.69,3)	4.98 (1.58,16)	3.91 (0.94,16)
Some college, no degree, or AA	0.77 (0.32,2)	1.08 (0.36,3)	0.53 (0.13,2)	0.55 (0.26,1)	1.14 (0.43,3)	0.7 (0.22,2)	0.54 (0.26,1)	1.13 (0.43,3)	0.71 (0.22,2)
Mater's degree, Professional or Doctorate Degree	0.87 (0.45,2)	0.96 (0.44,2)	1.48 (0.54,4)	0.83 (0.48,1)	0.95 (0.47,2)	1.52 (0.64,4)	0.83 (0.48,1)	0.93 (0.46,2)	1.57 (0.66,4)
<$30,000		1.36 (0.33,6)	3.27 (0.58,18)		0.95 (0.31,3)	1.77 (0.42,7)		0.95 (0.31,3)	1.74 (0.41,7)
$30,000-$49,999		0.78 (0.2,3)	1.14 (0.19,7)		0.78 (0.26,2)	1.06 (0.24,5)		0.75 (0.25,2)	1.06 (0.25,5)
$50,000-$74,999		1.48 (0.5,4)	2.4 (0.62,9)		1.55 (0.57,4)	1.97 (0.54,7)		1.52 (0.55,4)	1.86 (0.52,7)
$75,000-$99,999		1.44 (0.49,4)	1.97 (0.48,8)		1.13 (0.41,3)	1.66 (0.43,6)		1.14 (0.41,3)	1.58 (0.41,6)
Marital status		1.19 (0.46,3)	1.45 (0.48,4)		1.65 (0.69,4)	1.5 (0.61,4)		1.71 (0.72,4)	1.48 (0.59,4)
**Child Characteristics**									

Child’s sex		1.7 (0.85,3)		1.75 (0.97,3)		1.71 (0.95,3)
Birth weight	1 (1,1)		1 (1,1)		1 (1,1)	

OR= Odds Ratio and CI = Confidence Interval. OR and CI values indicated in bold presents those with significance values greater than .008. References groups for categorical variables were White (maternal race), college degree (maternal education), Hispanic (maternal ethnicity), and female (child’s sex).

Table S3. Results of Univariate Models Testing For the Effects Of COVID-Related Hardships on Change in ASQ Z-Scores.

	Communication		Gross motor		Fine Motor		Problem-Solving		Personal-Social	
	OR (95%CI)	P value	OR (95%CI)	P value	OR (95%CI)	P value	OR (95%CI)	P value	OR (95%CI)	P value
Parents' job loss/change	1.14 (0.72,2)	0.58	0.88 (0.53,1)	0.62	0.72 (0.42,1)	0.24	1.13 (0.58,2)	0.72	1.11 (0.64,2)	0.72
less contact with household members	0.92 (0.52,2)	0.78	0.8 (0.43,1)	0.49	1.35 (0.74,2)	0.33	0.58 (0.25,1)	0.18	0.77 (0.37,2)	0.48
Less contact with family/friends outside the home	1.64 (0.79,3)	0.18	0.85 (0.43,2)	0.64	0.66 (0.34,1)	0.21	0.92 (0.39,2)	0.85	1.22 (0.56,3)	0.61
Baseline ASQ Z-score	0.84 (0.67,1)	0.11	0.87 (0.66,1)	0.32	0.77 (0.6,1)	0.04	0.79 (0.58,1)	0.14	0.82 (0.63,1)	0.12
Maternal race (Reference: white)										
American Indian or Alaska Native	1.16 (0.51,3)	0.73	1.26 (0.56,3)	0.58	0.87 (0.35,2)	0.76	1.74 (0.7,4)	0.23	1.02 (0.42,2)	0.97
Asian	0.86 (0.45,2)	0.64	1.01 (0.5,2)	0.98	1.23 (0.6,3)	0.56	1.58 (0.66,4)	0.30	1.59 (0.79,3)	0.20
Black	1.47 (0.58,4)	0.42	1.66 (0.69,4)	0.26	1.44 (0.37,6)	0.60	2.12 (0.56,8)	0.27	1.09 (0.31,4)	0.90
Multiple race	1.55 (0.66,4)	0.32	0.39 (0.09,2)	0.20	0.88 (0.25,3)	0.84	1.33 (0.37,5)	0.67	1.5 (0.56,4)	0.42
Native Hawaiian or other Pacific Islander	4.57 (0.93,22)	0.06	3.24 (0.58,18)	0.18	14 (1.22,160)	0.03	5.25 (0.45,62)	0.19	3.62 (0.33,39)	0.29
Other race	2.59 (1.6,4)	0.00	0.77 (0.38,2)	0.47	1.09 (0.58,2)	0.79	1.53 (0.71,3)	0.28	1.08 (0.55,2)	0.82
Maternal ethnicity - Not Hispanic or Latino vs Hispanic	0.43 (0.29,1)	0.00	1.09 (0.67,2)	0.74	0.85 (0.52,1)	0.52	0.63 (0.36,1)	0.12	0.84 (0.51,1)	0.49

Maternal Education (Reference: college degree)										
Less than high school	2.81 (1.35,6)	0.01	1.11 (0.42,3)	0.83	3.24 (1.45,7)	0.00	3.15 (1.18,8)	0.02	0.85 (0.3,2)	0.76
High school degree, GED or equivalent	2.87 (1.57,5)	0.00	1.55 (0.76,3)	0.23	2.77 (1.3,6)	0.01	3.99 (1.7,9)	0.00	1.21 (0.55,3)	0.64
Some college, no degree, or AA	1.37 (0.75,2)	0.31	0.53 (0.25,1)	0.09	0.85 (0.36,2)	0.70	0.87 (0.33,2)	0.78	0.78 (0.37,2)	0.50
Mater's degree, Professional/Doctorate Degree	0.71 (0.41,1)	0.23	0.86 (0.5,1)	0.60	0.98 (0.49,2)	0.96	1.31 (0.59,3)	0.51	1.11 (0.61,2)	0.74
Maternal employment: No vs Yes	1.18 (0.46,3)	0.72	1.38 (0.62,3)	0.43	1.24 (0.36,4)	0.72	1.77 (0.66,5)	0.25	0.86 (0.28,3)	0.78
Household income (Reference: > $100,000										
<$30,000	3.04 (1.87,5)	0.00	1.2 (0.68,2)	0.53	1.83 (0.98,3)	0.06	2.45 (1.21,5)	0.01	1.21 (0.68,2)	0.52
$30,000-$49,999	1.68 (0.79,4)	0.18	0.71 (0.27,2)	0.49	1.37 (0.54,3)	0.50	1.36 (0.45,4)	0.59	1.17 (0.5,3)	0.72
$50,000-$74,999	2.06 (0.99,4)	0.05	0.83 (0.35,2)	0.68	1.58 (0.63,4)	0.33	1.63 (0.49,5)	0.43	1.44 (0.55,4)	0.45
$75,000-$99,999	1.1 (0.47,3)	0.82	1.13 (0.49,3)	0.77	1.28 (0.54,3)	0.58	1.15 (0.34,4)	0.82	0.82 (0.32,2)	0.68
Marital status	1.29 (0.69,2)	0.42	1.23 (0.6,2)	0.57	1.77 (0.83,4)	0.14	1.83 (0.81,4)	0.15	0.84 (0.34,2)	0.71
Preterm (37+wks vs < 37wks)	0.91 (0.47,2)	0.78	1.01 (0.46,2)	0.98	0.77 (0.39,2)	0.47	1.13 (0.42,3)	0.81	1.92 (0.79,5)	0.15
Children sex: Male vs Female	2.44 (1.61,4)	0.00			1.32 (0.81,2)	0.26	1.58 (0.9,3)	0.11	2.4 (1.46,4)	0.00
Children race (Reference: white)			0.78 (0.5,1)	0.26						

Black or African American	1.32 (0.52,3)	0.56	1.18 (0.43,3)	0.75	1.28 (0.27,6)	0.75	1.54 (0.36,7)	0.56	1.04 (0.27,4)	0.95
Others	0.8 (0.51,1)	0.33	0.88 (0.53,1)	0.61	0.89 (0.52,2)	0.67	1.26 (0.7,2)	0.44	1.04 (0.61,2)	0.87
Children's ethnicity: Not Hispanic vs Hispanic	0.42 (0.28,1)	0.00	1.17 (0.72,2)	0.52	0.78 (0.48,1)	0.30	0.66 (0.37,1)	0.15	0.93 (0.57,2)	0.76
Gestational age	0.98 (0.92,1)	0.59	0.97 (0.91,1)	0.45	1.01 (0.93,1)	0.83	1.05 (0.91,1)	0.53	1.1 (0.97,1)	**0.13**
Birth weight	1 (1,1)	0.85	1 (1,1)	**0.17**	1 (1,1)	0.28	1 (1,1)	0.37	1 (1,1)	0.21
Children Pre Pandemic Life Stage	0.79 (0.54,1)	0.23	1.19 (0.76,2)	0.44	1.05 (0.65,2)	0.83	1.42 (0.81,2)	0.22	0.66 (0.4,1)	**0.10**

Note. OR= Odds Ratio and CI = Confidence Interval; ASQ (Ages and Stages Questionnaire)

Table S4. Results of Multivariate Models Testing For The Effects Of COVID-Related Hardships on ASQ domain z-score change (adjusted for pre-pandemic ASQ Scores and Significant Covariates (p-values <.2).

	**Parents' job loss/change**			**Less contact with household members**			**Less contact with family/friends outside home**		
	Gross Motor	Fine motor	Problem solving	Gross Motor	Fine motor	Problem solving	Gross Motor	Fine motor	Problem solving
	OR (95%CI)	OR (95%CI)	OR (95%CI)	OR (95%CI)	OR (95%CI)	OR (95%CI)	OR (95%CI)	OR (95%CI)	OR (95%CI)
Parents' job loss/change	0.13 (-0.03,0.29)	0.16 (-.02,0.34)	0 (-.18,0.19)						
Less contact with household members				0.08 (-0.1,0.26)	0.04 (-.19,0.26)	0.22 (0.01,0.42)			
Less contact with family/friends outside home							0 (-0.22,0.22)	0.02 (-.23,0.28)	-0.05 (-0.3,0.2)
Baseline ASQ	-0.68 (-0.8,-0.55)	-0.65 (-0.74,-0.55)	-0.72 (-0.82,-0.61)	-0.71 (-0.81,-0.6)	-0.64 (-0.74,-0.55)	-0.65 (-0.75,-0.56)	-0.71 (-0.81,-0.6)	-0.64 (-0.74,-0.55)	-0.65 (-0.74,-0.56)
**Maternal Characteristics**									
American Indian or Alaska Native	-0.01 (-0.41,0.39)	0.34 (-0.04,0.73)	-0.22 (-0.68,0.24)	-0.1 (-.45,0.24)	0.34 (-.01,0.69)	-0.12 (-0.54,0.29)	-0.08 (-0.42,0.26)	0.35 (0,0.69)	-0.06 (-.46,0.33)
Asian	0.26 (0.03,0.49)	0.15 (-0.12,0.43)	0 (-0.28,0.27)	0.22 (0.02,0.41)	0.12 (-0.13,0.38)	-0.04 (-0.3,0.21)	0.22 (0.02,0.42)	0.13 (-0.13,0.38)	-0.02 (-0.27,0.24)
Black	-0.3	0.06	-0.07	-0.23	0.01	-0.05	-0.23	0.01	-0.05

	(-.76,0.16)	(-0.36,0.48)	(-0.58,0.44)	(-0.66,0.2)	(-0.45,0.47)	(-0.54,0.45)	(-0.66,0.2)	(-0.45,0.47)	(-0.55,0.45)
Multiple race	0.01	-0.1	-0.3	0.14	0.21	-0.03	0.14	0.22	-0.01
	(-.32,0.34)	(-0.53,0.34)	(-0.71,0.1)	(-0.13,0.41)	(-0.16,0.59)	(-0.38,0.33)	(-0.13,0.41)	(-0.16,0.59)	(-0.36,0.34)
Native Hawaiian or other Pacific Islander	-0.64	-0.68 (- 1.87,0.5)	-0.86	-0.6	-0.5	-0.57	-0.6	-0.51	-0.57
	(-.52,0.23)		(-2.35,0.64)	(-1.46,0.26)	(-1.61,0.61)	(-2.02,0.88)	(-1.46,0.26)	(-1.62,0.6)	(-2.02,0.89)
Other race	0.12	0.21	0.04	0.04	0.29	0.08	0.04	0.29	0.09
	(-.21,0.45)	(-0.17,0.6)	(-0.35,0.42)	(-0.24,0.32)	(-0.09,0.67)	(-0.27,0.42)	(-0.23,0.32)	(-0.09,0.67)	(-0.26,0.44)
Maternal ethnicity	0.06	0.05		0.09	0.11		0.09	0.11	
	(-0.17,0.3)	(-0.23,0.32)		(-0.11,0.3)	(-0.14,0.36)		(-0.11,0.3)	(-0.13,0.36)	
Less than high school	-0.3	Less than high school	-0.3	Less than high school	-0.3	Less than high school	-0.3	Less than high school	-0.3
High school degree, GED or equivalent	-0.07	-0.36	-0.11	-0.06	-0.38	-0.29	-0.06	-0.38	-0.3
	(-0.44,0.31)	(-0.75,0.03)	(-0.58,0.36)	(-0.38,0.25)	(-0.76,-0.01)	(-0.72,0.14)	(-0.38,0.25)	(-0.76,0)	(-0.74,0.13)
Mater's degree, Professional or Doctorate Degree	0.04	0.08	-0.08	0.07	0.1	-0.07	0.06	0.1	-0.08
	(-0.15,0.24)	(-0.13,0.29)	(-0.3,0.13)	(-0.11,0.24)	(-0.08,0.29)	(-0.26,0.12)	(-0.11,0.23)	(-0.08,0.28)	(-0.27,0.11)
Some college, no degree, or AA	0.13	-0.04	0.26	0.18	-0.01	0.13	0.18 (- 0.02,0.38)	-0.01	0.14
	(-0.1,0.35)	(-0.34,0.26)	(-0.04,0.55)	(-0.02,0.38)	(-0.26,0.25)	(-0.14,0.41)		(-0.26,0.25)	(-0.14,0.42)
<$30,000	-0.1		<$30,000	-0.1		<$30,000	-0.1		<$30,000
$30,000-$49,999	-0.04		-0.06	0.08			0.08		-0.02
	(-0.4,0.32)		(-0.41,0.28)	(-0.21,0.37)		0 (-0.4,0.39)	(-0.21,0.36)		(-0.42,0.37)
$50,000-$74,999	-0.07		-0.22	-0.04		-0.21	-0.04		-0.21
	(-0.32,0.19)		(-0.6,0.17)	(-0.3,0.21)		(-0.62,0.2)	(-0.3,0.21)		(-0.62,0.2)
$75,000-$99,999	-0.22		-0.2	-0.13		-0.13	-0.13		-0.12

	(-0.49,0.06)		(-0.53,0.13)	(-0.38,0.12)		(-0.43,0.17)	(-0.38,0.12)		(-0.43,0.18)
**Child Characteristics**									
Gestational age	0<br>(-0.03,0.04)		-0.02<br>(-0.05,0.02)	0.01<br>(-0.03,0.04)		-0.03<br>(-0.06,0)	0.01<br>(-0.03,0.04)		-0.02<br>(-0.06,0.01)
Preterm			-0.34<br>(-0.76,0.08)			-0.13<br>(-0.54,0.28)			-0.13<br>(-0.54,0.29)
Birthweight		0 (0,0)	0 (0,0)		0 (0,0)	0 (0,0)		0 (0,0)	0 (0,0)
Children pre-pandemic life stage		0.16<br>(-0.01,0.34)			0.15<br>(-0.02,0.33)			0.16<br>(-0.02,0.33)	
Child ethnicity			0.04<br>(-0.2,0.28)			0.08<br>(-0.15,0.31)			0.08<br>(-0.14,0.31)

Notes. OR= Odds Ratio and CI = Confidence Interval. OR and CI values indicated in bold presents those with significance values greater than .008.

References groups for categorical variables were White (maternal race), college degree (maternal education), Hispanic (maternal ethnicity), and female (child's sex).
